# The rise of electrochromics through dynamic QR codes and grayscale images in screen printed passive matrix addressed displays

**DOI:** 10.1038/s41598-022-14792-9

**Published:** 2022-07-08

**Authors:** Peter Andersson Ersman, Kathrin Freitag, Jun Kawahara, Jessica Åhlin

**Affiliations:** 1 Division Digital Systems, Department Smart Hardware, Units Printed, Bio- and Organic Electronics, RISE Research Institutes of Sweden, Norrköping, Sweden; 2 Lintec Corporation, Saitama, Japan

**Keywords:** Electrical and electronic engineering, Imaging and sensing

## Abstract

Electronic matrix addressed displays capable of presenting arbitrary grayscale images typically require complex device architectures including switching components to provide unique pixel addressability. Here, we demonstrate high-yield manufacturing of passive matrix addressed electrochromic displays on flexible substrates by solely using screen printing. The simple pixel architecture, obtained by printing only three active layers on top of each other, concurrently provides both the electrochromic functionality and the critical non-linear pixel switching response that enables presentation of arbitrary grayscale images in the resulting passive matrix addressed displays. The all-printed display technology exhibits unprecedented performance and is further verified by dynamic QR codes, to exemplify utilization within authentication, packaging, or other emerging Internet of Things applications requiring a low-cost display for data visualization.

## Introduction

In the strive towards Internet of Things (IoT) applications, often targeting sensor systems capable of ubiquitous monitoring of various parameters, there is also an increased demand for the ability to present the monitored results on an electronic display. However, targeting billions of IoT devices inevitably implies certain requirements of such electronic display technology, where unique pixel addressability in matrix configurations along with high-yield manufacturing at low cost are two of the most critical parameters. Various optoelectronic technologies are coexisting, and they may be used to create electronic displays that are operated in emissive, reflective or transmissive mode^1–4^. The active materials in these devices may be either organic or inorganic, and the operation mechanism relies on, *e.g.*, electroluminescence^5–7^, orientation of liquid crystals^8^, electrowetting^9^, or electrochromism^10^. Display segments, or pixels, are created through various patterning techniques, and the resulting display is updated either through direct addressing or by using a matrix configuration. However, forming a matrix addressed display by arranging the pixels in rows and columns generates cross-talk effects, *i.e.*, coloration of non-addressed pixels, and additional switching components are needed to obtain unique pixel addressability^11–15^. In addition to this, currently existing display technologies are relying on a combination of different manufacturing techniques, most often resulting in increased production costs.

**Figure 1:**
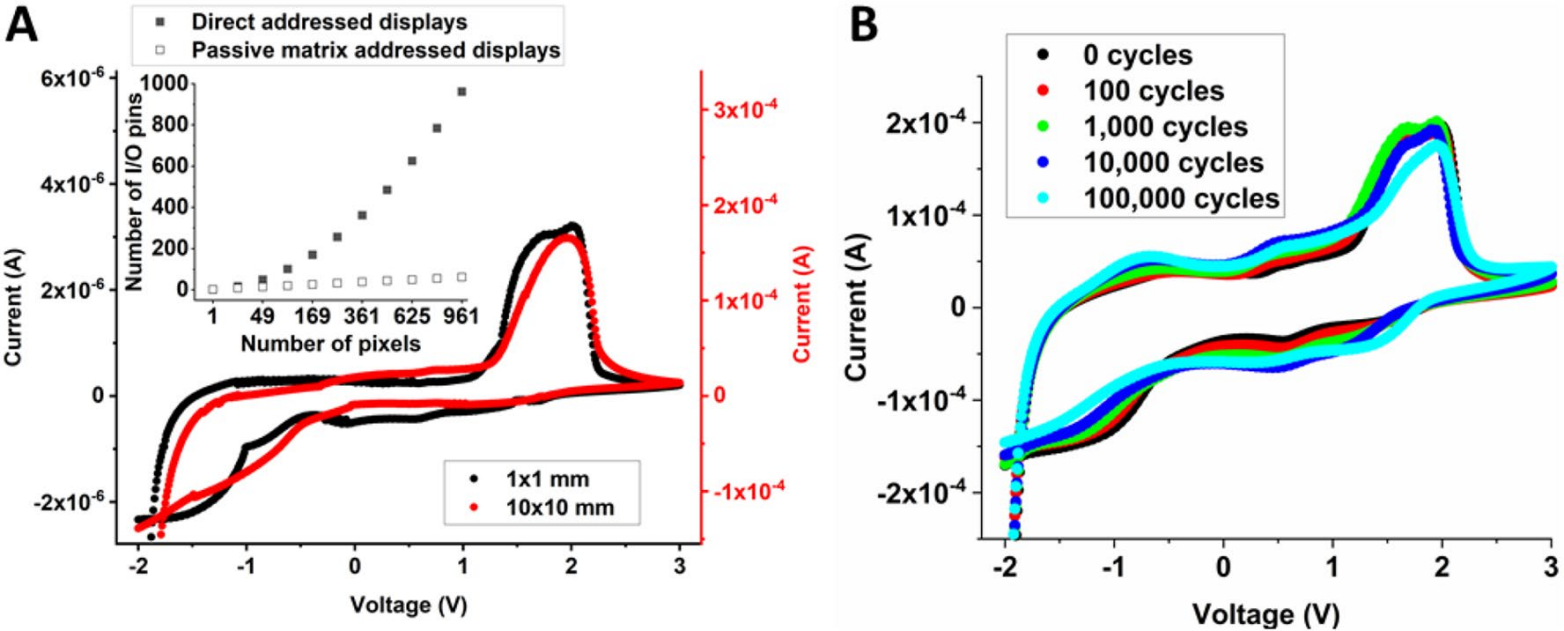
Non-linear pixel switching response obtained for various pixel areas, independent of the number of switching cycles. (**A**) The current vs. voltage characteristics show almost identical threshold voltages for two different pixel areas; 1 × 1 and 10 × 10 mm. The inset shows the benefit of using the passive matrix addressing protocol as compared to direct addressing. The number of I/Os required from the addressing electronics scales linearly with the number of pixels in direct addressed displays (filled squares), while the number of I/Os required in PMADs equals the sum of the rows and columns (open squares). The number of pixels on the x-axis corresponds to square-shaped pixel arrangements (7 × 7, 13 × 13, 19 × 19, 25 × 25 and 31 × 31). (**B**) Current vs. voltage curves recorded after a certain number of switching cycles (represented by the different colors), ranging from the initial switching behavior up to 10^5^ switching cycles.

The research field of printed electronics, often involving organic molecules or polymers as active materials in the components, is rapidly evolving. The technology implies novel form factors on flexible substrates by taking advantage of the solution processability of the active materials. Numerous publications are reporting on the development and manufacturing of individual components, and their integration into more complex systems, where printing techniques are used to deposit the materials from ink formulations^16–19^. Fully screen printed electrochromic displays, in which the segments are predefined and directly addressed, have been developed and matured during the last decade^20^, and these displays are now being commercialized by others^21^. The technology relies on poly(3,4-ethylenedioxythiophene) chemically stabilized by poly(styrene sulfonic acid) (PEDOT:PSS)^22^, which not only serves as the electrochromic material but also exhibits sufficiently high conductivity to obviate the need of indium; a rare earth element often found in the transparent conductive oxide coatings used in many commercial optoelectronic devices^23^. However, despite being a mature printed electronic component, the segments of screen printed electrochromic displays are direct addressed, thereby limiting the number of applications since each segment requires a unique input/output (I/O) channel in the external addressing circuit. Naturally, the concept of passive matrix addressed displays (PMAD) is a better alternative, where the pixel arrangement along the rows and columns of the matrix implies a substantial reduction in the number of required I/O channels, see inset in Fig. 1A.

Since PMADs are updated by row and column addressing, a non-linear pixel switching response is an immediate prerequisite to ensure display updating sequences without cross-talk between neighboring pixels^11^. This criterion was fulfilled in a previous report by using a non-reliable manufacturing approach including a mixture of printing, coating and lamination techniques, thereby resulting in PMADs completed by a lamination step to sandwich a non-cured electrolyte between the electrodes, which in turn limited the PMAD operation to a very narrow environmental condition window^24^. Here, we are instead reporting on PMADs manufactured by solely using screen printing on single flexible plastic substrates, thereby enabling a large variety of IoT applications requiring an electronic display capable of presenting arbitrary images, such as dynamic QR codes. In addition to this, the possibility to control the grayscale levels by adjusting amplitudes and/or pulse lengths of the applied voltages is also demonstrated. The required non-linear switching response, which enables the unique pixel addressability in PMADs, is obtained by screen printing only three active materials on top of each other (Supplementary Figs. S1 and S2): PEDOT:PSS (electrochromic layer), electrolyte (to facilitate electrochromic switching) and a carbon-based counter electrode layer.

## Results and discussion

### Current versus voltage characteristics for different pixel areas

To find the most optimized combination of materials, individual electrochromic pixels were screen printed by using different sets of ink formulations. The non-linear pixel switching response was evaluated by monitoring the electrochromic activity upon sweeping the voltage applied to the respective pixel, as shown by the current (I) vs. voltage (V) behavior in Fig. 1A. Positive voltages correspond to reduction of PEDOT:PSS to its blue colored state, while negative voltages result in oxidation of PEDOT:PSS to its transparent state. Note that in these displays the transparent PEDOT:PSS state corresponds to a white color due to that a white and opaque electrolyte is used, hence, the displays are operating in reflective mode. The most promising combination of materials resulted in reduction and oxidation of the PEDOT:PSS-based pixel electrode initialized at approximately 1 V and -0.5 V, respectively. By properly tuning the voltage amplitudes of the passive matrix addressing protocol, such threshold voltages are sufficiently high to enable updating of PMADs without cross-talk effects. Additionally, the evaluation was carried out for two pixel areas, differing by a factor of 100 (1 × 1 and 10 × 10 mm), which resulted in two different sets of current levels but yet approximately the same threshold voltages for both pixel areas. This is an important result indicating that PMADs with arbitrary pixel areas can be established, and proper update sequences can be achieved by using the same addressing protocol, independent of pixel area.

### Current versus voltage characteristics for different number of switching cycles

Cyclability is another important feature since this provides an estimation of the operational lifetime. The cycling test was performed on 1 × 1 mm pixels by applying a ± 3 V square wave pulse at a frequency of 10 Hz. Supplementary Videos S1 and S2 indicate that the pixel switching time is shorter than 50 ms, since the reduction and oxidation pulses provided by the 10 Hz square wave signal are sufficiently long to fully switch the pixels between their two coloration states. The initial I vs. V behavior, as well as after 10^2^, 10^3^, 10^4^ and 10^5^ switching cycles, are shown in Fig. 1B. All of the tested pixels were functional after 10^5^ switching cycles, even though minor degradation could be observed by comparing the consecutive measurements. An important result of this test is that the threshold voltage remained almost the same, independent of the number of switching cycles, which implies that the same voltage amplitudes can be used in the passive matrix addressing protocol throughout the operational lifetime of the PMAD.

### Arbitrary grayscale PMAD images enabled by the color contrast versus voltage characteristics

**Figure 2:**
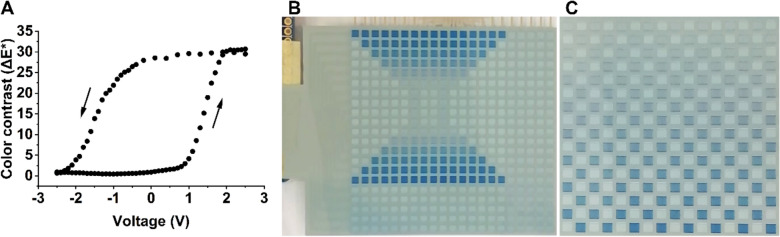
The color contrast (ΔE*) vs. voltage hysteresis enables arbitrary grayscale images in screen printed PMADs. (**A**) The graph shows that the desired hysteresis is obtained also when investigating the color contrast (ΔE*) vs. voltage. Upon exceeding the onset of the respective electrochromic switching event (1 V in the forward direction and -0.5 V in the reverse direction), the coloration scales almost linearly with the applied voltage, which in turn allows for the presentation of PMAD images with different grayscale levels. (**B**) The photograph shows a PMAD updated into an image resembling the shape of an hourglass. Besides the pixel addressability, eight different grayscale levels are also demonstrated in this image. (**C**) The photograph shows when the same PMAD instead is updated into a 16 × 16 chessboard pattern. Besides the ability to update the pixels of the PMAD without any discernable cross-talk effects, thereby allowing for the update of any kind of pattern in these screen printed electrochromic displays through passive matrix addressing protocols, 17 grayscale levels are also demonstrated by subsequently increasing the voltage amplitude in the row by row update sequence of the PMAD.

Since the objective of this work is to develop an electronic display device capable of presenting its content to the human eye, it is also important to investigate the correlation between the applied voltages, the threshold voltages (shown in Fig. 1) and the perceived color contrast. 10 × 10 mm pixels were therefore switched throughout a wide voltage window, to ensure reaching the full color contrast range. The measurement started at -2.5 V (fully oxidized white state). The voltage was gradually increased to 2.5 V, in steps of 100 mV, resulting in the fully reduced blue colored state. The process was then reversed by gradually decreasing the applied voltage until the fully oxidized state was reached again at -2.5 V. Each one of the consecutively increased/decreased voltage amplitudes was applied for ~ 10 s, to ensure saturation of the respective oxidation state, followed by the determination of the ΔE* color contrast value by measuring the CIE L*a*b* color coordinates of the pixel^25^. The results of this measurement are shown in Fig. 2A, where the arrows indicate whether the applied voltage was gradually increased (ΔE* exceeding 30 when reaching the fully reduced state of PEDOT:PSS at ~ 2 V) or decreased (oxidation from blue to white). The data correlate very well with the previously described I vs. V switching behavior. In the forward direction, the onset of the electrochromic switch occurs at ~ 1 V, while the reverse direction results in an onset at approximately  − 0.5 V; such hysteresis is desired and allows for passive matrix addressing. In addition to this, once exceeding the onset of the electrochromic switch, both switching directions show an almost linear dependence between the recorded color contrast and the applied voltage. This is yet another important result that enables grayscale imaging, or bluescale imaging due to the color range of PEDOT:PSS; a minor change in the voltage amplitude results in a corresponding change in the color contrast perceived by the human eye. The ability to present arbitrary grayscale images is shown in Figs. 2B and C, wherein the same PMAD is used to present two subsequently updated images with up to 17 discrete grayscale levels. The complete PMAD update sequences are shown in Supplementary Videos S3 and S4.

### Dynamic QR codes

**Figure 3:**
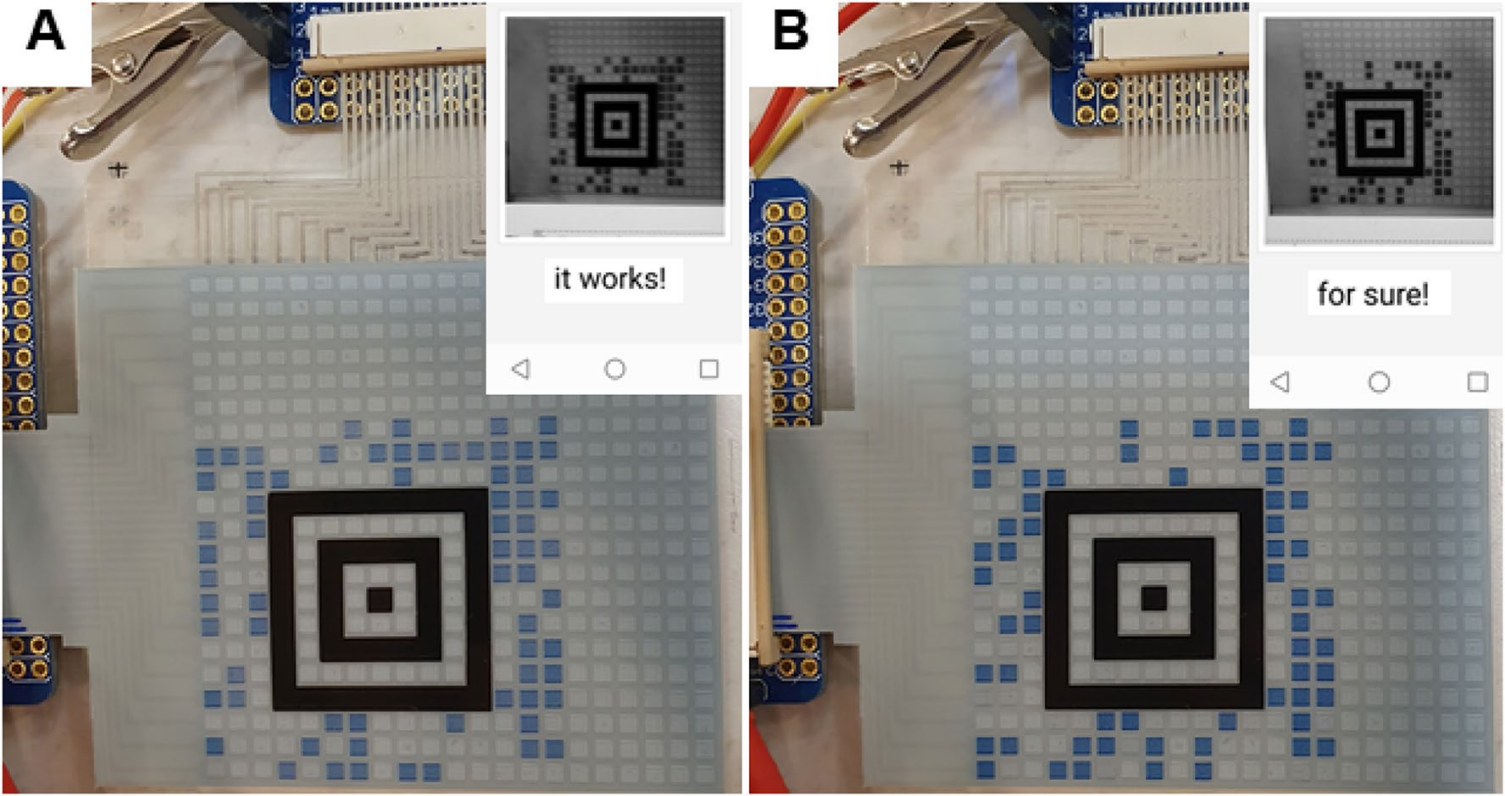
The concept of dynamic QR codes allows for novel IoT applications, *e.g.*, within authentication. Two different QR codes are subsequently presented in the same PMAD device, resulting in two unique messages upon scanning the images with a mobile phone; (**A**) *it works!* and (**B**) *for sure!*. The insets are screenshots stored by the mobile phone, showing both the recorded QR code images and the interpreted messages.

To further emphasize on the ability to present any arbitrary image in the screen printed PMADs, and to verify the possibility of utilization in emerging IoT applications, the concept of dynamic QR codes is demonstrated in Fig. 3. PMADs with 21 × 21 and 29 × 29 pixels were screen printed for this purpose. However, due to the limitation of the external addressing electronic circuit, which only contains 32 channels, the generation of dynamic QR codes had to be limited to Aztec codes containing 15 × 15 pixels, thereby also limiting the amount of information that can be stored in each image. But nevertheless, by installing an Aztec code reader on a mobile phone, it was possible to scan and read the messages *it works!* and *for sure!* in the respective image presented in Fig. 3. The complete update sequences of the dynamic QR codes are provided in Supplementary Videos S5 and S6. Note that Aztec codes are commonly used in ticketing systems, *e.g.*, to provide electronic boarding passes in the airline industry^26^.

### Manufacturing yield and pixel fill factor

The simple device architecture of the electrochromic pixels allows for high manufacturing yield of the resulting PMADs, despite that the relatively rough screen printing technique is used in every processing step. The design layout of the screen printing tools covers approximately an A4 area and generates a total of 3006 pixels, separated into 12 PMADs and 2 segment-based displays with various layouts and pixel dimensions. The manufacturing yield was evaluated by switching all pixels in two different sheets originating from the same print batch, see Fig. S3. Fully operational pixels exhibit long retention time (~ 50% color retention after 40 min), see Fig. S4, which imply that malfunctioning pixels suffering from electrical short-circuits are easily detected. Using this visualization approach resulted in a pixel manufacturing yield of at least 99.8%. Hence, the screen printing process results in remarkably reliable PMAD manufacturing.

The pixel fill factor, defined as the switchable pixel area vs. the total area required for each pixel, is dependent on the targeted pixel area of the PMAD. The smaller pixels areas, the more difficult to obtain high pixel fill factor. Here, in most of the evaluated PMADs, the openings in the insulating layer provide a switchable pixel area of 1.9 × 1.9 mm, and the total area required for each pixel is 2.54 × 2.54 mm, which corresponds to a pixel fill factor of 56%. By instead targeting a total pixel area of 10 × 10 mm, while using the same set of design rules with 0.64 mm separation between two neighboring pixels, would result in a fill factor of ~ 88%, thereby enabling large-area electronic billboard applications.

### PMAD addressing protocols

**Figure 4:**
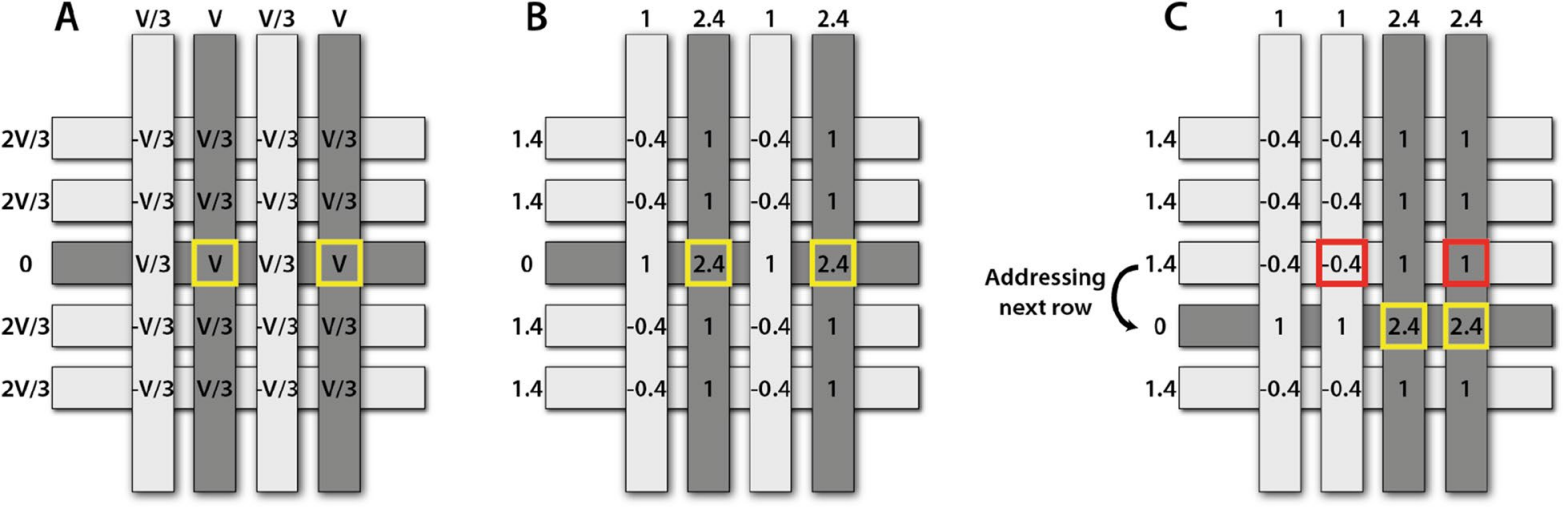
The V/3 addressing protocol is used to update screen printed PMADs, here exemplified by a 5 × 4 display architecture. (**A**) Strictly following the V/3 protocol results in ± V/3 in non-addressed PMAD pixels, which in turn would generate cross-talk effects for the pixel architecture presented herein. (**B**) An optimized addressing protocol, still relying on the V/3 addressing method, is used to update the PMADs. The voltage amplitudes are skewed due to the non-symmetric pixel switching response; approximately 1 V and -0.5 V is required to initialize the reduction and oxidation of the PEDOT:PSS-based pixel electrodes, respectively. (**C**) The PMADs are updated row by row, and the schematic illustrates a part of an updating sequence when shifting from row 3 to row 4. As an example, the pixels in columns 2 and 4 are updated when row 3 is addressed, as indicated by the red squares in row 3. Once this has been completed, the row addressing is shifted to the subsequent row, and an arbitrary number of pixels can be updated in the currently addressed row, here exemplified by applying the voltage V (2.4 V) to columns 3 and 4. The colored pixels in previously addressed rows retain their color since the resulting voltages in non-addressed pixels remain below the threshold voltages. The yellow squares in the schematics illustrate pixels currently being addressed. The currently addressed row and columns are indicated by dark gray color in the schematics.

A data acquisition card (DAQ-card) provides the voltage amplitudes required to obtain PMAD operation without cross-talk effects. Each row and column of the PMAD is connected to a respective channel on the DAQ-card, this is a requirement since open-circuit mode operation inevitably would generate cross-talk effects. The V/2 and V/3 addressing protocols are both feasible through optimization of the applied voltage amplitudes^24^. The non-linearity of the pixel switching response eventually defines the amplitude levels that should be applied to the addressed and non-addressed pixels of the PMAD to minimize cross-talk, *i.e.*, the resulting voltages sensed by non-addressed pixels should remain below the threshold voltage levels of the electrochromic onset. Here, a modified version of the V/3 addressing protocol is used to present the intended PMAD images at maximum color contrast between addressed and non-addressed pixels. 2.4 V is applied to the column of an addressed pixel to switch it to its fully colored state, while 0 V is applied to the corresponding row. Ideally, to strictly follow the V/3 addressing protocol, 0.8 V and 1.6 V should be applied to non-addressed columns and rows, respectively. However, this would generate a symmetric voltage of ± 0.8 V applied to the non-addressed pixels, see Fig. 4A. The current vs. voltage curves shown in Fig. 1A and the color contrast vs. voltage curve shown in Fig. 2A clearly demonstrate non-symmetric threshold voltages, with a current/(de)coloration onset at approximately 1 V and -0.5 V for the respective direction of the electrochromic switch. Hence, strictly following the V/3 addressing protocol would result in cross-talk, since -0.8 V clearly exceeds -0.5 V. Instead, a skewed version of the V/3 addressing protocol was developed to minimize cross-talk effects. Many different combinations of voltage amplitude levels have been evaluated, and to apply 0 V to the addressed row, 1.4 V to non-addressed rows, 1.0 V to non-addressed columns and 2.4 V to addressed columns seems to be one of the most promising set of voltages, see Fig. 4B. This implies that the resulting voltages in non-addressed pixels become either 1.0 V or -0.4 V; two voltage amplitudes that are below, or in worst case equal to, the threshold voltages of the electrochromic pixels.

The PMADs are updated row by row, hence, an arbitrary number of columns can be addressed for each particular row selection. Once the content of one row has been successfully updated, the subsequent row is instead selected by shifting the row addressing voltage (0 V), see Fig. 4C.

To use 2.4 V as the switching voltage to obtain full color contrast could be argued. Figure 2A indicates that the color contrast is saturated at ~ 2 V in the forward direction. Hence, from a cross-talk perspective, it would be wise to lower the maximum voltage to this value. However, this particular measurement was performed at steady state, *i.e.*, each one of the gradually increased/decreased voltage levels was applied for a long time (~ 10 s) to ensure saturation of each discrete color contrast value. The maximum color contrast, the pixel switching time and the degree of PMAD cross-talk are ultimately governed by the maximum switching voltage, and the 2.4 V that was used throughout this report was considered to be the best trade-off, *i.e.*, high color contrast and short pixel switching time with maintained pixel addressability.

Grayscale imaging can be achieved by two different methods. One option is to obtain discrete color contrast levels by using short pulse lengths when applying the voltages to the rows and columns, as demonstrated by a row scanning time of ~ 40 ms in Supplementary Video S6. This implies a gradually appearing image upon continuously cycling the rows. Full color contrast is achieved after ~ 10 complete PMAD update sequences, indicating that at least the same number of grayscale levels are accessible. Note that an even shorter row scanning time would result in an increased number of grayscale levels. The other option is instead to adjust the voltage amplitudes to obtain the grayscale imaging effect, and this method is used in the images presented in Fig. 2. The image in Fig. 2B shows eight different grayscale levels, they are obtained by adjusting the voltage applied to the column of an addressed pixel, in this case by gradually decreasing/increasing (200 mV steps) the voltage from 1 V (white colored state) to 2.4 V (dark blue colored state) in the subsequently updated rows. In Fig. 2C, the 17 different grayscale levels are achieved by relying on the same voltage range (1 to 2.4 V), but the step between two consecutive rows upon increasing the voltage applied to the column of an addressed pixel is instead ~ 50 mV. The voltages of addressed rows (0 V), non-addressed rows (1.4 V) and non-addressed columns (1.0 V) remain the same. It should be noted that different row scanning times were used to obtain the images in Fig. 2B and C. The updating sequence shown in Supplementary Video S3, which is linked to Fig. 2B, is based on a short row scanning time of ~ 40 ms, thereby demonstrating that many more grayscale levels become accessible by using a combination of adjustable row scanning times and adjustable voltage amplitudes. The prolonged row scanning time (500 ms) in the updating sequence shown in Supplementary Video S4, which is linked to Fig. 2C, instead provide grayscale levels obtained solely by the adjustable voltage amplitudes.

In summary, this work demonstrates full addressability, without noticeable cross-talk between neighboring pixels, in electrochromic PMADs manufactured on flexible substrates by solely using screen printing. The pixels exhibit short switching times (~ 50 ms), great cyclability (> 10^5^ switching cycles) and high color contrast (ΔE* ~ 30), and the desired non-linear pixel switching response is easily obtained by stacking only three active materials on top of each other; the PEDOT:PSS-based electrochromic material, the electrolyte and the carbon-based counter electrode, thereby allowing for sheet by sheet or roll to roll low-cost production at high manufacturing yield (~ 99.8%). Hence, this strongly indicates the robustness of the screen printed PMAD technology presented herein. In addition to the achievements of switching performance, robustness and non-linear pixel switching response, the possibility to switch each pixel into at least 17 different grayscale levels is also demonstrated. Together, the findings allow for a holistic approach ranging from high-yield manufacturing of PMADs by screen printing to the presentation of arbitrary grayscale images for a variety of IoT applications, *e.g.*, electronic shelf labels, dynamic QR codes within authentication and packaging, entertainment board games, large-area electronic billboards, or within distributed healthcare by visualizing data from ubiquitous monitoring in (bio)sensor platforms.

## Methods

### PMAD architecture

The screen printed electrochromic PMADs presented herein are operated in reflective mode. The electrochromic layer is firstly deposited on the surface of the plastic substrate, and the color change is therefore observed through the plastic film. Hence, the substrate not only serves as a carrier, it also provides mechanical protection of the printed layers. A white opaque electrolyte is utilized to hide the counter electrode and to enable operation in reflective mode. Supplementary Figs. S1 and S2 illustrates the PMAD architecture and the screen printing process, respectively. Optionally, additional graphical and/or protection layers may be deposited, but for simplicity they are omitted in the schematics.

### Manufacturing process

All ink materials, see Supplementary Fig. S2, were deposited by a flatbed, sheet-fed, screen printing equipment (*DEK Horizon 03iX*) on top of flexible plastic (PET) substrates. The screens used in this work were based on standard polyester mesh. The materials in layers (1), (2), (5) and (6) were thermally cured; 120 °C for ~ 2 min in a hot air conveyor belt oven. The materials in layers (3) and (4) were UV-cured.

### Optical and electrical characterization

Optical and electrical characterization have been performed to evaluate the screen printed electrochromic pixels and PMADs. A spectrophotometer (*Mercury, Datacolor*) was used to determine the color contrast. The measurements provide the CIE L*a*b* color coordinates as well as the ΔE* color contrast when comparing the color coordinates obtained for PEDOT:PSS switched to different oxidation states; this approach is commonly used in the graphical industry and has also been reported previously for reflective electrochromic displays^25^. Characterization of the current vs. voltage switching behavior, to determine the non-linear pixel switching response, was performed by using a semiconductor parameter analyzer (*HP/Agilent 4155B*). Continuous pixel cycling was carried out by using a function generator (*Agilent 33120A*). PMAD operation was performed by using a DAQ-card (*NI PCI-6723*) capable of providing up to 32 unique analog voltage amplitudes. Characterization and evaluation of pixels and PMADs were performed at a temperature of ~ 20 °C and a relative humidity level of ~ 45–50%RH.

## Supplementary Information

Supplementary Information 1.

Supplementary Video 1.

Supplementary Video 2.

Supplementary Video 3.

Supplementary Video 4.

Supplementary Video 5.

Supplementary Video 6.

## Data Availability

The data that support the plots within this article and other findings of this study are available from the corresponding author upon reasonable request.
