# Supplementary material for: The rise of electrochromics through dynamic QR codes and grayscale images in screen printed passive matrix addressed displays
Source: Sci Rep. 2022 Jul 8;12:10959. doi: 10.1038/s41598-022-14792-9 (PMC9270494; doi:10.1038/s41598-022-14792-9)
Supplement: Supplementary file 1 — Supplementary Information 1. [file 41598_2022_14792_MOESM1_ESM.docx]

**Supplementary information**

**The rise of electrochromics through dynamic QR codes and grayscale images in screen printed passive matrix addressed displays**

Peter Andersson Ersman*, Kathrin Freitag, Jun Kawahara & Jessica Åhlin

* Corresponding author: peter.andersson.ersman@ri.se

**The Supplementary information includes:**

Supplementary Figs. S1-S4

Captions for Supplementary Videos S1 to S6


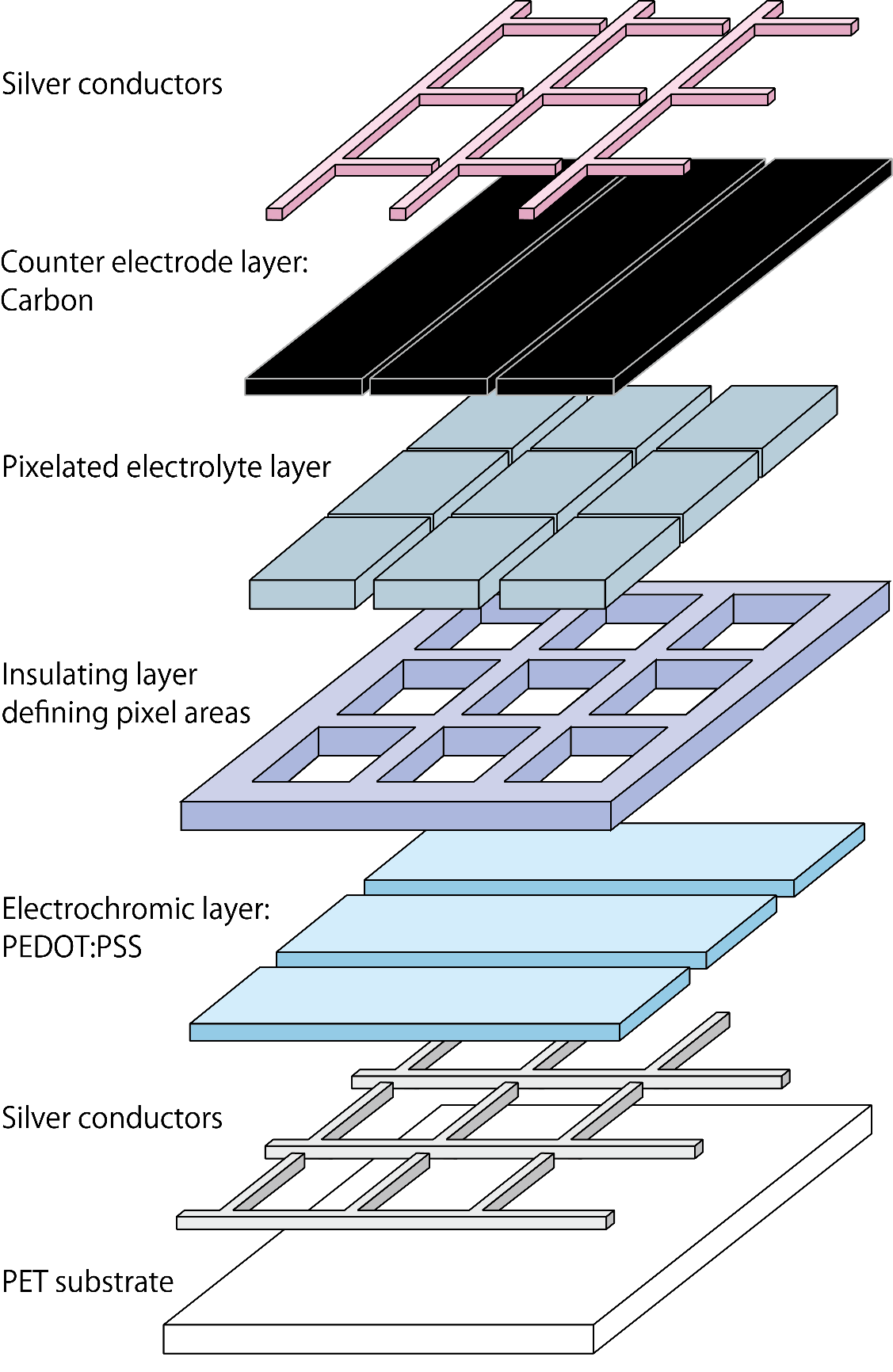


**Supplementary Fig. S1. Schematic showing a 3×3 PMAD architecture.** The electrochromic switching of addressed pixels is observed through the plastic substrate, which thereby provides mechanical protection of the screen printed PMAD.


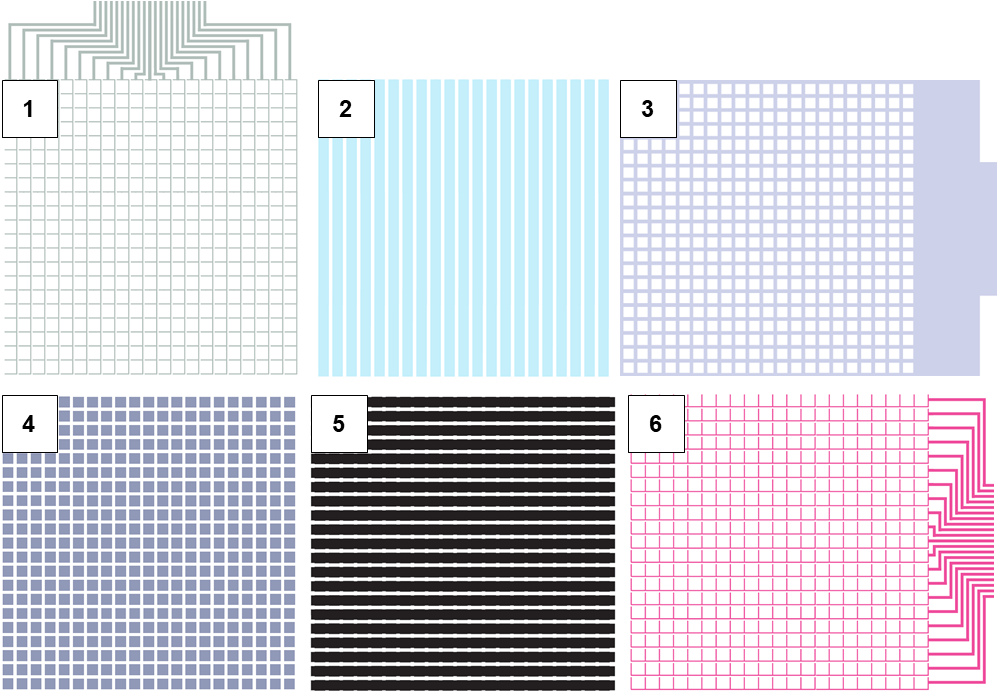


**Supplementary Fig. S2. The sequence of the manufacturing process, here exemplified by a 21×21 PMAD.** All materials are deposited by screen printing on top of a flexible plastic (PET) substrate. **1** Silver (*CXT 0644*, *Sun Chemical*) conductors to provide contact pads for the external addressing electronic circuit and to minimize resistive loss along the subsequently deposited electrochromic stripes. **2** PEDOT:PSS (*Clevios S V4*, *Heraeus*) stripes that serve as the electrochromic color changing electrodes. **3** Insulating (*UVSF*, *Marabu*) layer that prevents electrical short-circuits and defines the shape and area of the respective electrochromic pixel. **4** Electrolyte (*E001*, *RISE*) layer that facilitates electrochromic switching. **5** Carbon (*7102*, *DuPont*) stripes serving as the counter electrodes. **6** Silver (*CXT 0644*, *Sun Chemical*) conductors to minimize resistive loss along the counter electrode stripes. 2, 4 and 5 are the active materials that provide the electrochromic functionality, and the vertical pixel architecture is formed in the respective row and column crossing, in which the electrolyte is sandwiched between the electrochromic PEDOT:PSS electrode and the carbon counter electrode.


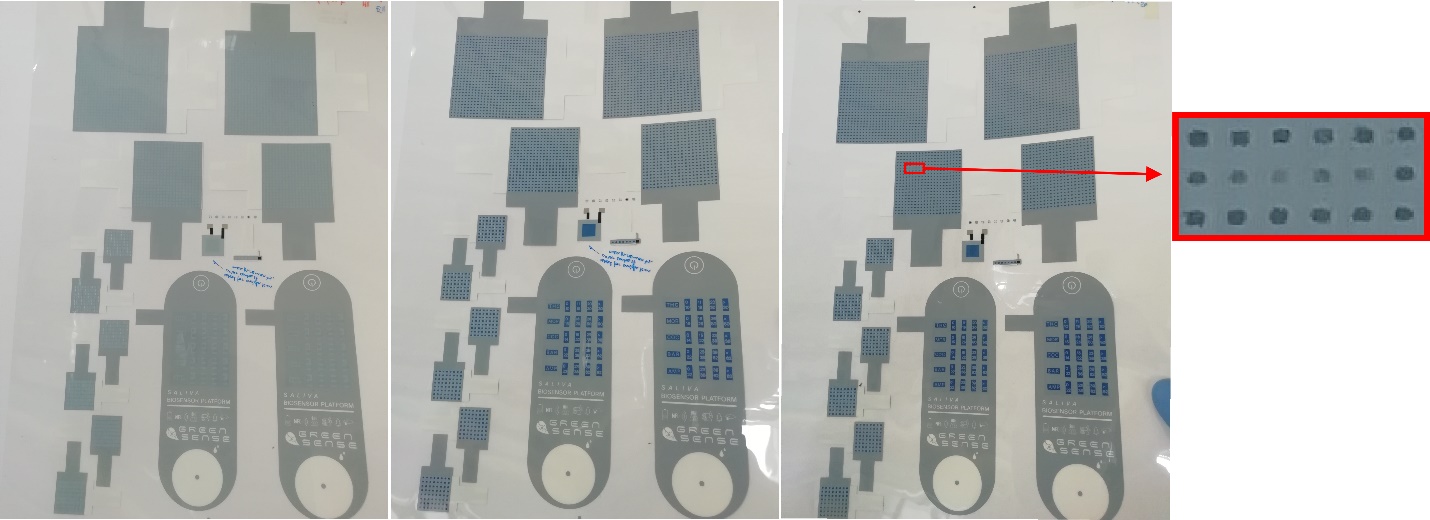


**Supplementary Fig. S3. The manufacturing yield evaluated from the pixels screen printed on two different substrates. Left:** All pixels were initially switched to the oxidized white state by applying -3 V to the counter electrodes. Each substrate contains 3006 pixels, spread out in 14 different displays of various layouts (12 PMADs and 2 segment-based) and dimensions. **Middle:** All pixels of this sheet were switched to the reduced blue colored state by applying 3 V to the counter electrodes. The pixels were then left in open-circuit mode for ~30 s, to allow any malfunctioning pixels to spontaneously discharge towards the white state. However, the blue color remained in all pixels printed on this sheet. **Right:** The procedure was repeated for the second sheet, which resulted in ~8-10 malfunctioning pixels, exemplified by 3-4 pixels exhibiting weaker color contrast in the center of the zoom-in image. Hence, in these two sheets, a pixel manufacturing yield of at least 99.8% was obtained, despite using the relatively rough manufacturing method of screen printing. It should be noted that all pixels were switching. Hence, from a functionality point of view, it would be possible to update all these PMADs by using a sufficiently high refresh pulse frequency.


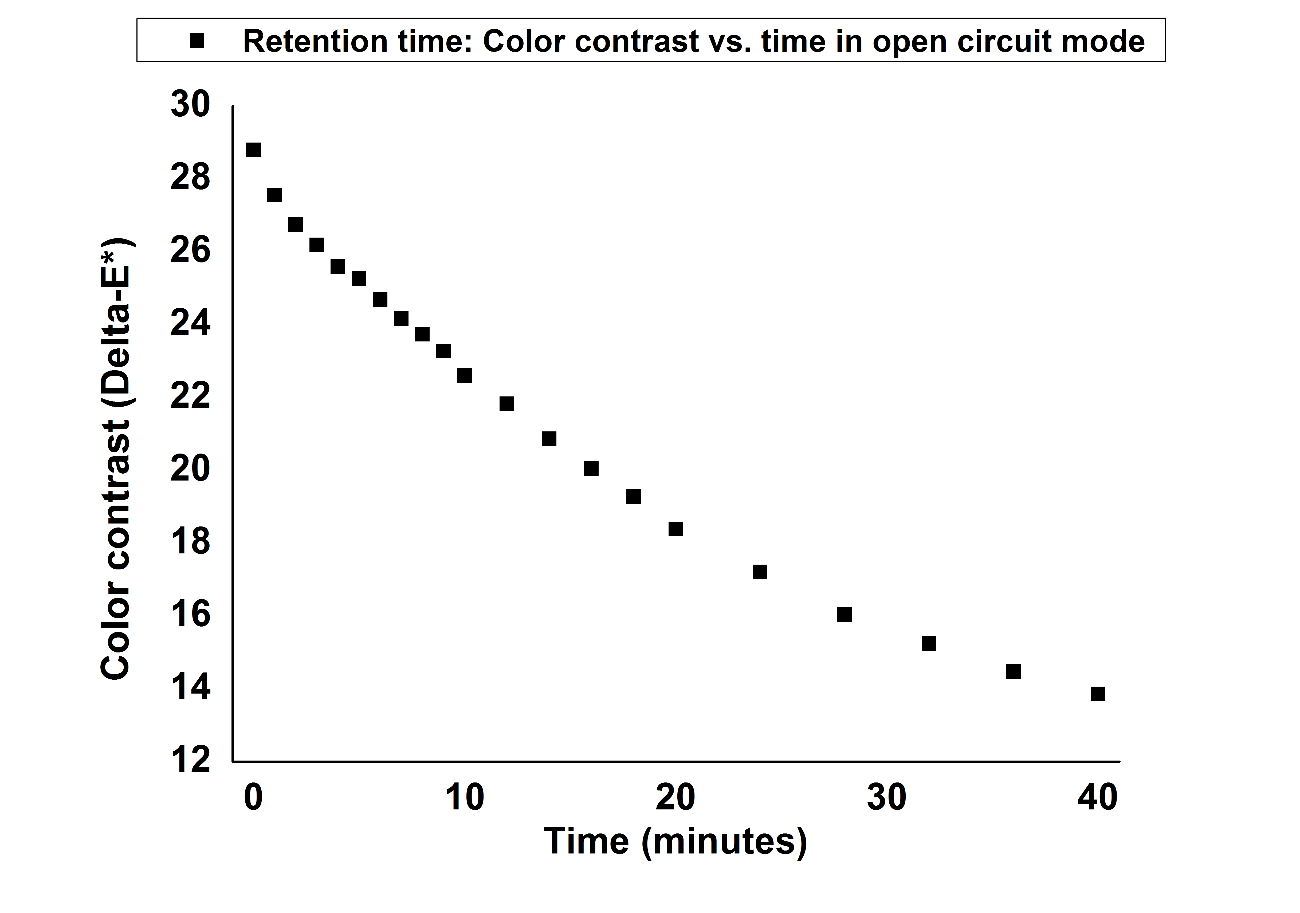


**Supplementary Fig. S4. The retention time of a fully functional pixel.** Initially, a 10×10 mm pixel is switched to its reduced blue colored state by applying 3 V to its counter electrode, thereby reaching a color contrast of ΔE*~29. The voltage is disconnected for the remainder of the measurement, *i.e.*, open-circuit mode, and the color contrast of the pixel is continuously measured. The color contrast has dropped to approximately 50 % of the initial value after 40 minutes. On the contrary, a malfunctioning pixel shows a much more rapid color fading behavior, typically resulting in a retention time of less than one minute.

**Supplementary Video S1. Pixel switching response shown in real time.** A ±3 V square wave voltage pulse is applied to an 8×8 PMAD at a frequency of 10 Hz. The movie shows the pixel switching in real time.

**Supplementary Video S2. Pixel switching response shown in slow motion.** Supplementary Video S1 slowed down ~15 times. This indicates that the maximum pixel switching time is 50 ms, since the pixels are reaching their fully oxidized and reduced states at a frequency of 10 Hz.

**Supplementary Video S3. The update sequence of a PMAD image resembling the shape of an hourglass with eight grayscale levels.** The following different voltage amplitudes are applied to the columns to obtain the image of an hourglass consisting of eight different grayscale levels: 1, 1.2, 1.4, 1.6, 1.8, 2, 2.2, 2.4 V. The video shows how the image gradually appears in the PMAD, and full color contrast is reached after ~10 complete PMAD addressing cycles, this is due to the high row scanning frequency (write time ~40 ms per row).

**Supplementary Video S4. Chessboard pattern with 17 grayscale levels.** The same PMAD used in Supplementary Video S3 is here updated into a new image of a 16×16 chessboard pattern including 17 different grayscale levels by applying the following voltages to the columns: 1, 1.4, 1.45, 1.5, 1.55, 1.6, 1.65, 1.7, 1.75, 1.8, 1.85, 1.9, 1.95, 2, 2.05, 2.1, 2.4 V. The row scanning frequency is lowered to 500 ms per row, such that only one complete row scan of the PMAD is needed to achieve full color contrast in the addressed pixels.

**Supplementary Video S5. Dynamic QR code demonstrated at low row scanning frequency.** Low row scanning frequency is used to update the PMAD by just using one complete addressing cycle. The message *it works!* is acquired when reading the resulting image with an Aztec code reader installed on a mobile phone.

**Supplementary Video S6. Dynamic QR code demonstrated at high row scanning frequency.** High row scanning frequency is used to update the image of the same PMAD shown in Supplementary Video S5. Full color contrast is reached after ~10 complete PMAD addressing cycles, but the message *for sure!* could be interpreted by the Aztec code reader on the mobile phone already after four complete PMAD addressing cycles. This verifies the concept of dynamic QR codes in screen printed electrochromic PMADs.
